# Nanoencapsulation of Phenolic Extracts from Native Potato Clones (*Solanum tuberosum* spp. *andigena*) by Spray Drying

**DOI:** 10.3390/molecules28134961

**Published:** 2023-06-24

**Authors:** Carlos A. Ligarda-Samanez, David Choque-Quispe, Elibet Moscoso-Moscoso, Henry Palomino-Rincón, Fredy Taipe-Pardo, John Peter Aguirre Landa, José C. Arévalo-Quijano, Jenny C. Muñoz-Saenz, Uriel R. Quispe-Quezada, Mary L. Huamán-Carrión, Edgar Gutiérrez-Gómez, Reynaldo Sucari-León, Rober Luciano-Alipio, Judy M. Muñoz-Saenz, Rodrigo J. Guzmán Gutiérrez

**Affiliations:** 1 Food Nanotechnology Research Laboratory, Universidad Nacional José María Arguedas, Andahuaylas 03701, Peru; elibetmm22@gmail.com; 2 Nutraceuticals and Biomaterials Research Group, Universidad Nacional José María Arguedas, Andahuaylas 03701, Peru; dchoque@unajma.edu.pe (D.C.-Q.); hpalomino@unajma.edu.pe (H.P.-R.); ftaipe@unajma.edu.pe (F.T.-P.); huamancarrionmary@gmail.com (M.L.H.-C.); 1008820181@unajma.edu.pe (R.J.G.G.); 3 Research Group in the Development of Advanced Materials for Water and Food Treatment, Universidad Nacional José María Arguedas, Andahuaylas 03701, Peru; 4 Agroindustrial Engineering, Universidad Nacional José María Arguedas, Andahuaylas 03701, Peru; 5 Water and Food Treatment Materials Research Laboratory, Universidad Nacional José María Arguedas, Andahuaylas 03701, Peru; 6 Agroindustrial Research Laboratory, Universidad Nacional José María Arguedas, Andahuaylas 03701, Peru; 7 Business Administration Department, Universidad Nacional José María Arguedas, Andahuaylas 03701, Peru; jpaguirre@unajma.edu.pe; 8 Department of Education and Humanities, Universidad Nacional José María Arguedas, Andahuaylas 03701, Peru; jcarevalo@unajma.edu.pe; 9 Human Medicine Faculty, Universidad Peruanalos Andes, Huancayo 12006, Peru; d.jmunoz@upla.edu.pe; 10 Agricultural and Forestry Business Engineering, Universidad Nacional Autónoma de Huanta, Ayacucho 05000, Peru; uquispe@unah.edu.pe; 11 Engineering and Management Faculty, Universidad Nacional Autónoma de Huanta, Ayacucho 05000, Peru; egutierrez@unah.edu.pe (E.G.-G.); rsucari@unah.edu.pe (R.S.-L.); 12 Administrative Sciences Faculty, Universidad Nacional Autónoma Altoandina de Tarma, Junín 12731, Peru; rluciano@unaat.edu.pe; 13 Environmental Technology Center, Servicio Nacional de Adiestramiento en Trabajo Industrial, Lima 15036, Peru; judmusa1@gmail.com

**Keywords:** optimization, response surface, central composite rotatable design, bioactive compounds, antioxidant capacity

## Abstract

Native potato clones grown in Peru contain bioactive compounds beneficial to human health. This study aimed to optimize the spray-drying nanoencapsulation of native potato phenolic extracts utilizing a central composite design and response surface methodology, obtaining the optimal treatment to an inlet temperature of 120 °C and an airflow of 141 L/h in the nano spray dryer B-90, which allowed maximizing the yield of encapsulation, antioxidant capacity (DPPH), encapsulation efficiency (EE), total phenolic compounds, and total flavonoids; on the other hand, it allowed minimizing hygroscopicity, water activity (Aw), and moisture. Instrumental characterization of the nanocapsules was also carried out, observing a gain in lightness, reddening of the color, and spherical nanoparticles of heterogeneous size (133.09–165.13 nm) with a negative ζ potential. Thermal, infrared, and morphological analyses confirmed the encapsulation of the core in the wall materials. Furthermore, an in vitro release study of phenolic compounds in an aqueous solution achieved a maximum value of 9.86 mg GAE/g after 12 h. Finally, the obtained nanocapsules could be used in the food and pharmaceutical industry.

## 1. Introduction

In the food and pharmaceutical industries, the loss of bioactive compounds during technological processes is recurrent, so it is necessary to study modern strategies to solve this problem. In this context, nanoencapsulation technology is commonly used to preserve and enhance the benefits of unstable or sensitive compounds. This process involves trapping one substance in another, producing nanoparticles of less than two microns [1,2]. The polymeric matrices protect the core against factors that could reduce their bioavailability and bioaccessibility [3,4].

Nanoaspersion drying is the most important alternative method for the nanoencapsulation of bioactive compounds worldwide. It is used because of its flexibility, cost-effectiveness, and versatility, as it does not affect the sensory characteristics or texture of the products. The formation of nanoparticles occurs in a single step in a continuous and scalable process, which makes it possible to obtain encapsulates at the nanometer level, enhancing their beneficial effect on human health [5,6,7,8,9]. The primary motivation for using this technology is to achieve a high yield with maximum encapsulation efficiency, considering that the main factors affecting it are the formulation and the operating parameters of the equipment [10]. Properly managing spray-drying conditions determines the final size of the nanocapsules [8], which overcome compatibility problems with the food matrix, affecting the quality of the final products. Moreover, nanoencapsulation allows designing a controlled and intelligent delivery of bioactive compounds at the gastrointestinal level [11].

Phenolic compounds are secondary metabolites of plants, and flavonoids containing three aromatic rings (C_6A_, C_3B_, and C_6C_) are the most common in the human diet. They are classified into anthocyanins, chalcones, flavones, flavanones, flavanols, and isoflavonoids; their interest is attributed to their human health benefits [12] and, more recently, to their possible antiviral potential against COVID-19 [13]. The bioavailability of flavonoids is generally low and could vary drastically between different classes and individual compounds of a particular group [14], so through biotechnology and genetic engineering, they seek to improve this problem. In addition, the industry seeks to extract phenolic compounds and stabilize them until consumption [15]. The low availability of flavonoids is associated with their poor gastrointestinal degradation and low distribution in food matrices [14,16].

Currently, mixtures of gum arabic and maltodextrin are being used in the nanoencapsulation of various compounds [17,18,19,20,21]. Gum arabic is obtained from *Acacia senegal* L. [22] and is an emulsifying biopolymer with low aqueous viscosity [23,24], so it is being used in nano spray-drying processes [25,26]. Maltodextrin is obtained from starch hydrolysis, is water soluble, tasteless, has low viscosity, and is available at affordable prices [17,27,28]. Mixing in different proportions of these wall materials has excellent results in nanoencapsulation for the food industry [17,27,29,30]. Nevertheless, polymeric matrices such as native starches, hydrocolloids, glucomannans, albumin, tara gum, tragacanth gum, and vinal gum are also used [30,31,32,33,34,35,36,37]. This study focused on improving the bioavailability of extractives from native potato clones, since the nanometer level of the particles would enhance their absorption in the gastrointestinal tract. It is known that several technologies have been used to stabilize bioactive compounds, such as nanoemulsions, nanoliposomes, nano spray drying, and electrospinning [2]. In addition, phenolic compounds with functional activities, such as quercetin, catechin, folic acid, thymol, resveratrol, and anthocyanins, were recently nanoencapsulated [18].

Therefore, the present study seeks to add value to these underutilized raw materials on account of higher field yields, high dry matter content, low levels of reducing sugars, and better nutritional and functional properties than other native potatoes grown in Peru and other countries in the Andean region of South America. Furthermore, this study hypothesized that inlet temperature and airflow influenced various properties during spray drying. The aim was to optimize the nanoencapsulation process of phenolic extracts from native potato clones in polymeric matrices of gum arabic and maltodextrin by varying the inlet temperature and airflow in the nano spray dryer B-90.

## 2. Results and Discussions

### 2.1. Characterization and Selection of the Best Native Potato Clone for Nanoencapsulation of Extracts

Table 1 shows the results of the properties studied. In the case of phenolic compounds, values between 4.57 and 6.72 mg gallic acid equivalent (GAE)/g were obtained, showing significant differences (*p* ≤ 0.05) in most of the clones; these results were within the range reported for native potato clones in Chile (1.92–18.64 mg GAE/g) [38]. In the case of flavonoids, values between 1.68 and 3.00 mg quercetin equivalent/g were reported, which were higher than those reported in New Zealand-colored potatoes (0.03 mg quercetin equivalent/g) [39] and lower than those reported in native Bolivian potatoes (19 mg quercetin equivalent/g) [40]. The antioxidant capacity DPPH was between 15.08 and 131.57 µmol Trolox equivalent (TE)/g, and the antioxidant capacity ABTS was between 17.52 and 22.83 µmol TE/g, values higher than those reported in 52 cultivars of native Andean potatoes (0.2 and 10 µmol TE/g) [40]. Finally, the anthocyanin content ranged from 2.53 to 7.79 mg Cyanidin-3-Glucoside (C3G) equivalent/g. The variability in the abovementioned properties is attributed to genotypic factors related to variety, agroecological conditions, and crop farming [26]. Furthermore, native potatoes contain bioactive compounds that prevent various degenerative diseases due to their high content of polyphenols, anthocyanins, flavonoids, carotenoids, and vitamins C, B_3_, and B_6_ [38,41,42,43,44,45]. Taking into account phenolic compounds, flavonoids, antioxidant capacity (DPPH and ABTS), and anthocyanins, clone 1 had the highest values, so it was chosen to develop the nanoencapsulation of its phenolic extracts in maltodextrin and gum arabic matrices.

### 2.2. Effect of Inlet Temperature and Airflow on Response Variables

Table 2 shows the 12 treatments considered and the experimental results of yield, hygroscopicity, water activity, moisture, antioxidant capacity by DPPH, encapsulation efficiency, phenolic compounds, and flavonoids obtained in the nanoencapsulation of the extracts of clone 1. Encapsulation yield values ranged from 66.28 to 74.28%, hygroscopicity from 7.82 to 12.26%, Aw from 0.35 to 0.45, moisture from 2.45 to 3.21%, antioxidant capacity from 1.11 to 28.38 µmol TE/g, encapsulation efficiency from 68.93 to 89.26%, phenolic compounds from 4.19 to 5.43 mg GAE/g and flavonoids from 0.6 to 2.18 mg quercetin/g. One of the main advantages of nanoencapsulation is the high content of bioactive compounds that can be encapsulated [46].

Table 3 shows the analysis of the variance (ANOVA) of the dependent variables of the rotatable composite central design developed. Below is the individual analysis for each response variable considered for optimization. The ANOVA results are used to fit the experimental values to the predicted mathematical model, considering the regression coefficient and the significance level [47].

#### 2.2.1. Yield of Encapsulation

**Figure 1:**
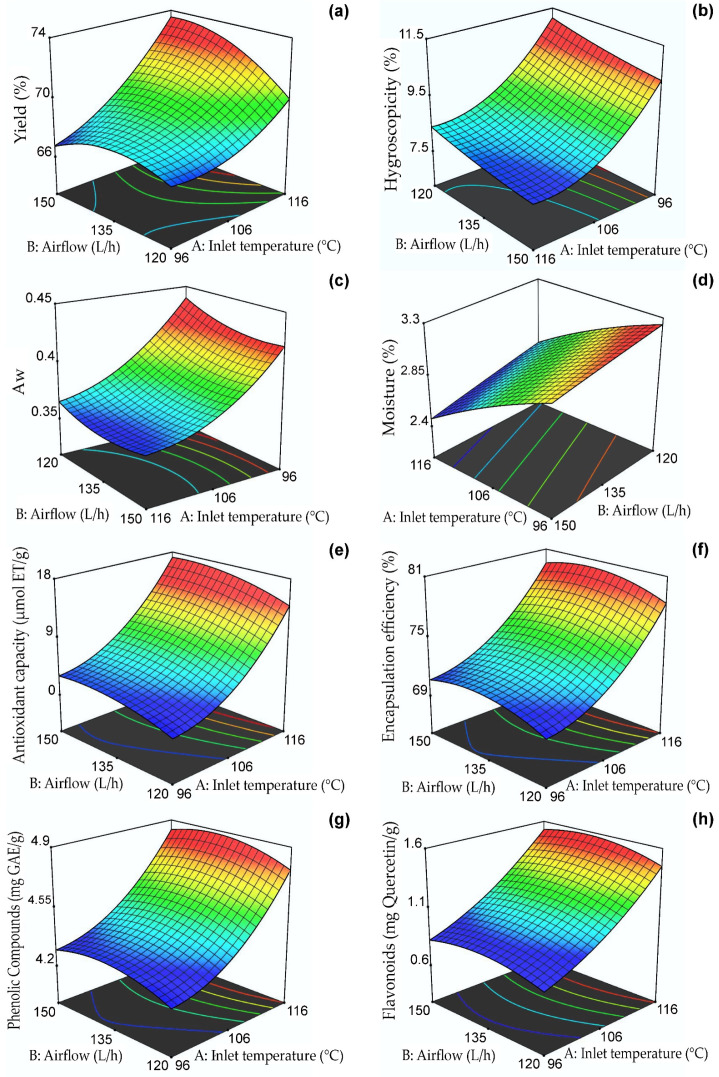
Response surfaces of the dependent variables: (**a**) yield, (**b**) hygroscopicity, (**c**) Aw, (**d**) moisture, (**e**) antioxidant capacity, (**f**) encapsulation efficiency, (**g**) phenolic compounds, and (**h**) flavonoids.

Table 3 shows that the yield obtained a regression coefficient R^2^ of 0.96, which indicates that the response surface model would explain 96% of the variability. Effects of inlet temperature and airflow were also observed in addition to the interaction of both variables and their quadratic models (*p* ≤ 0.05). The yield response surface, as presented in Figure 1a, indicates that the highest yield values (between 66 and 74%) were observed at temperatures ranging from 111–116 °C and flow rates of 135–150 L/h. It was observed that an increase in inlet temperature led to an increase in yield due to the loss of water, which made it easier to obtain powders in the collection cylinder of the nano spray dryer B-90 [35]. Similar values were reported for oxalis extracts microencapsulated in taro starch [48], purple-fleshed potato extracts microencapsulated in maltodextrin [49], and purple corn extracts microencapsulated in phosphorylated starch [50]. According to recent research reports, encapsulation performance is also affected by the soluble solids content of the spray-dried mixture [47,51]. The powder yield was used to determine the efficiency of the process, demonstrating that the use of a constant combination of maltodextrin/arabic gum carriers allowed obtaining high results, higher than 50%, which is considered a limit value in this type of process; on the other hand, the losses would be attributed to the adherence in the drying chamber.

#### 2.2.2. Hygroscopicity

Hygroscopicity is an essential property for the proper preservation of dehydrated foods (should be < 20%) and is defined as the capacity to absorb water from its surroundings [35,47]. Table 3 shows its coefficient of determination R^2^ of 0.97; effects of inlet temperature, airflow, and the quadratic temperature model were also observed (*p* ≤ 0.05). Figure 1b shows the response surface for hygroscopicity between 7.5 and 11.5%, with the lowest values between 106–116 °C and 135–150 L/h. The encapsulates at higher temperatures and higher air flow presented low values of hygroscopicity due to the formation of a compact surface on the nanocapsules that prevented trapping external water vapor [52]. The results were lower than those reported for spray-dried purple potato extract microcapsules (33.6%) [49] and were similar to those reported by Ligarda et al. [35]. The hygroscopicity was affected by the amount of encapsulant used due to the presence of hydroxyl groups in maltodextrin and gum arabic. In addition, the nanometer particle size favored water absorption due to the larger surface contact area [47,53]. According to the results, the nanocapsules would have good stability during storage.

#### 2.2.3. Water Activity

Aw is a property that allows determining the availability of water to participate in different deterioration mechanisms. Values below 0.6 allow the adequate preservation of dehydrated products, although it is recommended that a value of 0.3 in Aw not be exceeded [31,35,37]. Table 3 shows that Aw obtained a regression coefficient R^2^ of 0.99. Effects of inlet temperature, airflow, and their quadratic models were observed (*p* ≤ 0.05). Figure 1c shows the response surface for Aw between 0.35 and 0.45, with the lowest values observed between 108–116 °C and 130–150 L/h. The Aw results were higher than those reported for oxalis extracts microencapsulated in taro starch (0.221) [48] and purple potato extract microcapsules (0.225) [49]. It is important to note that values lower than 0.48 can prevent deterioration when storing nanocapsules for an extended time [28]. The high inlet temperatures could explain these results, which generated a thermal gradient and a consequent decrease in Aw. Other variables that could affect this property are the encapsulant type and the phenolic extracts’ chemical composition [48].

#### 2.2.4. Moisture

Moisture percentage is vital in dehydrated products and serves to understand their shelf life since it is closely related to glass transition and crystallization properties [54]. Table 3 shows that moisture obtained a regression coefficient R^2^ of 0.91. Effects of inlet temperature and airflow (*p* ≤ 0.05) were also noticed. Figure 1d shows the response surface for humidity between 2.4 and 3.3%, with the lowest values observed between 114–116 °C and 135–150 L/h. The values obtained in the present study ranged between 2.45–3.21% and were lower than those reported for saffron extracts nanoencapsulated in maltodextrin, which were between 3.28 and 3.92% [28]. On the other hand, they were similar to those reported by Yinbin et al. [55]. Variations in moisture content could be attributed to the chemical composition of the wall materials and the presence of hydrophilic groups in gum arabic and maltodextrin, which could act with the water molecules during storage of the nanocapsules [48]. In addition, moisture was affected by the type of carrier and drying temperature, resulting in dry powders of less than 5% water which, combined with a high production yield, has a positive effect on this product’s shelf life, packaging, and microbiological stability.

#### 2.2.5. Antioxidant Capacity DPPH

Antioxidant activity assays are used to study polyphenols’ free radical scavenging capacity in nanocapsules [34]. Table 3 shows that this property presented a regression coefficient R^2^ of 0.82. On the other hand, a significant effect of the inlet temperature variable was observed (*p* ≤ 0.05). Figure 1e displays the antioxidant capacity response surface for values ranging from 2 to 18 µmol TE/g. The highest values were found to occur at temperatures between 114–116 °C and airflows of 138–150 L/h. It is known that the protection and bioavailability of bioactive compounds in encapsulated products depend on the inlet temperature, amount of core, and proportion of wall materials used [48,56].

#### 2.2.6. Encapsulation Efficiency

Figure 1f shows the effect of the independent variables on the EE of phenolic compounds, which was between 68.93 and 89.26% and whose highest value corresponded to treatment T6 (120 °C and 135 L/hr), which would be related to the formation of an external layer of gelatinized starch at a high temperature, which would limit the release of the polyphenols present in the core [48]. This high EE would confirm the good retention capacity of phenolic compounds using the maltodextrin/gum arabic mixture [35]. Similar results were reported in microencapsulated blueberry extracts [57] and microcapsules of purple corn extracts [58]. Table 3 shows that the inlet temperature significantly affected the EE (*p* ≤ 0.05) with an R^2^ coefficient of determination of 0.71 in the model. The encapsulation efficiency was affected by the addition of the carrier and the incomplete extraction of phenolic compounds due to the strong interaction of functional components and wall materials.

#### 2.2.7. Total Phenolic Compounds

At higher drying temperatures, the polyphenol content increases due to the almost immediate formation of a crust on the surface of the nanocapsules, resulting from the acceleration of the drying rate in the droplets, which avoids heat loss [48]. Another property affecting phenolic compounds’ content is viscosity, which was 15–17 cP, so an increase in total solids will improve the polyphenol content [59]. Table 3 shows that phenolic compounds obtained a regression coefficient R^2^ of 0.71; the effect of inlet temperature (*p* ≤ 0.05) on the dependent variable was also observed. Figure 1g shows the response surface for phenolic compounds between 4.2 and 4.9 mg GAE/g, with the highest values observed between 114–116 °C and 138–145 L/h. The results were higher than those reported for microencapsulated purple rice extracts (0.43–1.04 mg GAE/g) [59] and lower than those reported for microencapsulated plum phenolic extracts by Yinbin et al. [55].

#### 2.2.8. Total Flavonoids

Flavonoids give color to vegetables and are part of the phenolic compounds. They play an essential role in plant growth due to their anti-pathogenic action [60]. Native potatoes are native to Peru and have antioxidant potential due to their high flavonoid content [61]. Table 3 shows that the total flavonoid content in the nanocapsules obtained a regression coefficient R^2^ of 0.71. The effect of inlet temperature (*p* ≤ 0.05) on the dependent variable was also observed.

Figure 1h shows the response surface for total flavonoid content between 0.6 and 1.6 mg quercetin/g, with the highest values observed between 114–116 °C and 130–140 L/h. Pedrozo et al. [34] produced nanocapsules of the flavonoid rutin and reported a decrease in its initial content due to the effect of the inlet temperature in the nano spray dryer B-90; similar behavior was observed in the present investigation.

#### 2.2.9. Optimization in the Nanoencapsulation of Extracts of the Selected Native Potato Clone

The fitted models of each response variable were used to determine the optimal nanoencapsulation treatment of the native potato clone extract, for which the desirability function multiple response analysis was used, which is a method that allows maximizing the results of the dependent variables with a score ranging from 0 to 1 [62]. Table 4 shows the regression coefficients of the mathematical models obtained.

Table 5 shows the optimum treatment, which consisted of an inlet temperature of 120 °C and airflow of 141 L/h with the desirability of 0.86, for obtaining the optimum nanoencapsulation conditions. Yield of encapsulation, antioxidant capacity, encapsulation efficiency, total phenolic compounds, and total flavonoids were maximized, while hygroscopicity, Aw, and moisture were minimized. The predicted values of the dependent variables were also obtained, which showed assertiveness compared to the results obtained experimentally.

### 2.3. Instrumental Characterization

#### 2.3.1. Color Analysis

Natural pigments present beneficial properties for health, so it is necessary to preserve them; the spray-drying method is the most used for this purpose [63]. Table 6 shows the results of color in the nanocapsules, observing that encapsulation increased the brightness (L*) and redness (a*) concerning the initial color of the atomized extract (L*: 17.09, a*: 1.62 and b*: −3.48), which would be attributed to the white color of the maltodextrin and gum arabic that were used as wall materials [64]. In addition, no noticeable changes in ΔE*_ab_ values were observed for most of the nanocapsules, which would be attributed to the proportions used of maltodextrin and gum arabic (90 and 10%) and their transparency in the solutions prepared before nano spray drying, which had an impact on the fact that no significant changes in color were observed. On the other hand, due to the effect of the high temperature, anthocyanin degradation occurred, which resulted in the formation of various polyphenols that influenced the final color of the nanocapsules.

Color is a property related to the quality of dehydrated products, so it is essential for food choice by consumers [52]. The inlet temperature in spray-drying processes has been shown to influence color variation [58]. Similar results were reported in pink microcapsules of plum phenolic extracts in maltodextrin and gum arabic [55], so the nanocapsules obtained in the present study could be used as a red colorant in functional foods and nutraceuticals.

#### 2.3.2. SEM Analysis

**Figure 2:**
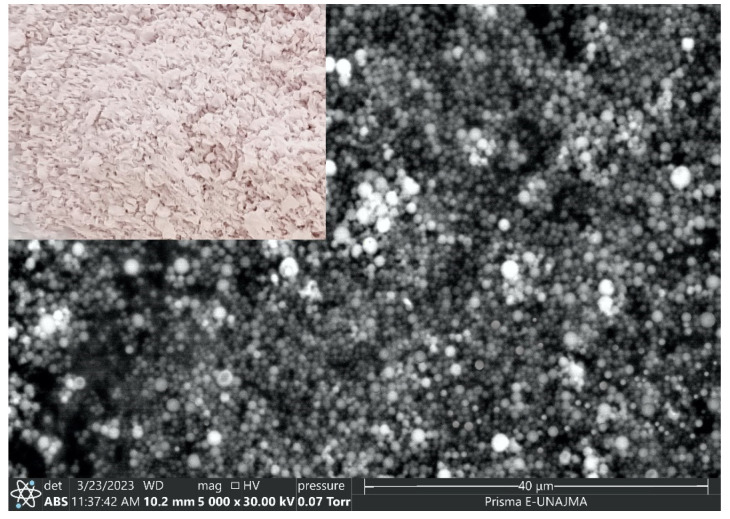
SEM micrograph of the optimal nanocapsule.

Figure 2 shows the micrograph of the optimum treatment, where spherical nanocapsules with smooth surfaces and variable sizes are observed; the size variation could be attributed to the differences in air flow in the B-90 nano spray dryer [65]. The measurements obtained by SEM were similar to those determined by dynamic light scattering (DLS) in the present study but less than that reported for nanocapsules of the flavonoid rutin in albumin between 200 and 600 nm dispersed in water [34]. The nanoparticles obtained in the nano spray dryer are characterized by having a broad size distribution, because it is complicated to control the parameters that determine the morphology and size in this equipment [35,66].

#### 2.3.3. Particle Size and ζ Potential

Table 7 shows the particle size of all the nanocapsules, which varied between 133.09 and 165.13 nm, showing that the particles presented a homogeneous distribution. Current studies in the pharmaceutical and food fields have demonstrated the advantages of nanoparticles (<200 nm) in in vivo and in vitro studies [34]. This size variation is attributed to the working conditions in the B-90 nano spray dryer (inlet temperature and airflow) [65]. The ζ potential is a measure that allows knowing the stability of the nanocapsules. This property is responsible for the electrostatic repulsion between particles, and values higher than ±30 mV in colloidal solutions would indicate a stable system since the particles reject each other [34]. The present study reported values between −22.79 and −31.12 mV (Table 6), indicating that the particles presented medium and slight stability. The changes in the ζ potential values would be attributed to the physical incorporation of the phenolic extracts into the wall materials used.

#### 2.3.4. FTIR Analysis

**Figure 3:**
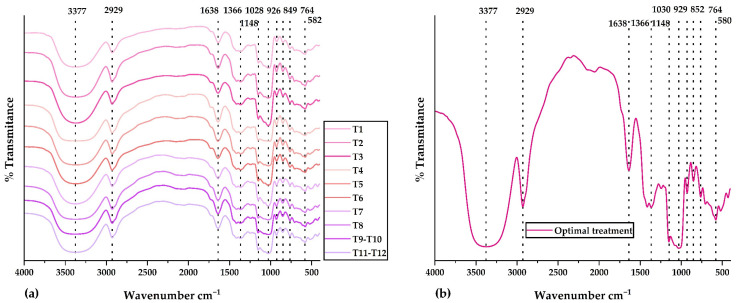
IR spectra in (**a**) all treatments of the central composite design and (**b**) optimal treatment.

Figure 3a displays the IR spectra for all treatments in the central composite design. The IR spectrum of the optimal treatment is observed in Figure 3b. Validated methodology and previously developed research were used for the FTIR analysis [35,37,67,68]. Similar functional groups were observed in all IR spectra. The peak at 3377 cm^−1^ would correspond to amino and hydroxyl groups present in polyphenols, glucids, polypeptide chains, and residual water. The peak at 2929 cm^−1^ would belong to amino acids and carboxylic acids (NH_3_ and CH functional groups). The peak at 1638 cm^−1^ would be attributed to phenolic compounds and flavonoids (carbonyl and ketone functional groups). The peak at 1028 cm^−1^ would be associated with extracts of phenolic compounds rich in ether, ester, alcohol, and carboxylic acid functional groups. The peaks below 929 cm^−1^ would be linked to the aromatic ring present in phenolic compounds and their structural modifications resulting from spray drying [35,37]. Research has shown that using the spray-drying technique can effectively shield the bioactive compounds within the nanocapsules and validate the encapsulation of the core in the wall materials. This finding is consistent with the results reported by Soto et al. [69] and Pashazadeh et al. [70]. The FTIR analysis determined that the nanoencapsulation developed from physically incorporating the phenolic extracts and the matrices. In addition, no significant alterations in intensity were observed since there were no chemical interactions.

#### 2.3.5. Thermal Analysis

**Figure 4:**
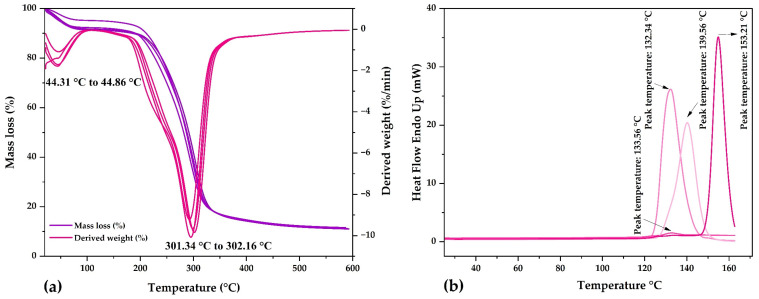
(**a**) TG and DTA in the nanocapsules and (**b**) DSC in the nanocapsules.

The TG and DTA curves were similar in all nanocapsules (Figure 4a), observing that a first event between 44.31 and 44.86 °C produced about a 4% mass loss, attributable to the onset of water loss that continues until temperatures are between 90 and 100 °C [71]. A second event occurred between 301.34 and 302.16 °C with a mass loss of about 60%. This high degradation rate, at around 300 °C, is attributable to the thermal decomposition of carbohydrates; after this temperature, thermal depolymerization of the biopolymers continues until their final volatilization [71]. According to these results, the nanocapsules have great thermal stability due to the use of maltodextrin and gum arabic as wall materials [35].

In the DSC thermograms of the nanocapsules (Figure 4b), endothermic peaks were observed at glass transition temperatures between 133.56 and 153.21 °C, similar to the glass transition temperature of maltodextrin (153.98 °C) [70,72,73] and spray-dried encapsulated corn polyphenols (143.40 °C) [70]. The glass transition temperatures of the nanocapsules were close to that of maltodextrin, which was the wall material used in the highest percentage (90%). The glass transition temperatures lower than that of maltodextrin confirm the encapsulation of the phenolic compound core, which formed inclusive complexes with the matrices used [70].

The glass transition temperatures reported in the present investigation would be attributed to different factors, such as chemical composition, molecular weights, and moisture contents. The interactions that occur between polyphenol-rich extracts and wall materials can be monitored by FTIR and DSC analysis. The reported endothermic peaks are due to the sum of glass transition and melting and are also attributable to the polysaccharide content conferred by using maltodextrin and gum arabic encapsulants.

### 2.4. Release of Polyphenols in Aqueous Solution

**Figure 5:**
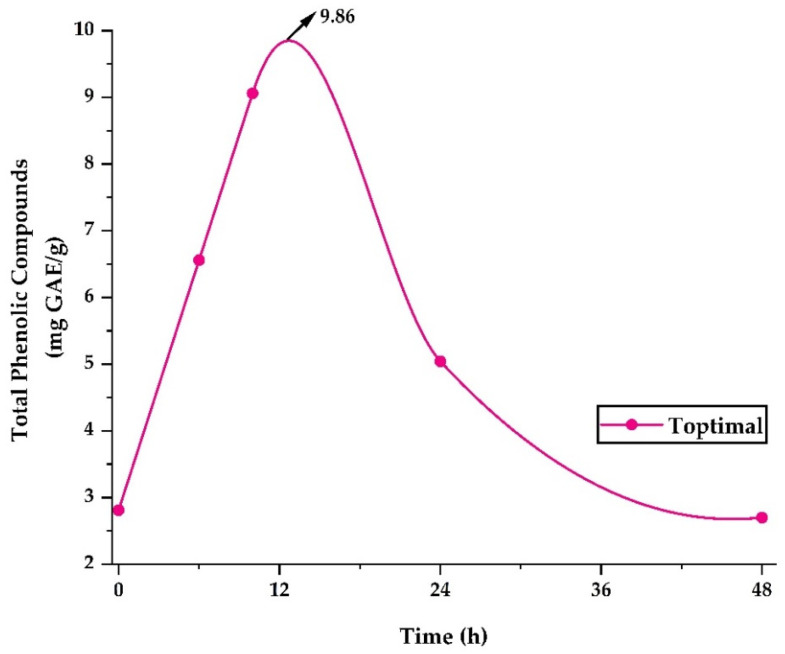
Release of phenolic compounds in the optimal treatment.

Figure 5 shows the release of phenolic compounds in the optimum treatment. The highest release of 9.86 mg GAE/g occurred after 12 h, after which a steady decrease was observed until 48 h. The study of polyphenol release allows an approach to their behavior in foods with high water content and to understand their possible behavior at the gastrointestinal level [35,37]. Nevertheless, it is necessary to carry out more in-depth stability studies in food products and bioaccessibility studies at the gastrointestinal level [74].

## 3. Materials and Methods

### 3.1. Materials

The native potato clones (*Solanum tuberosum* spp. *andigena*) were harvested in October 2022 and were kindly provided by engineer José Palomino Flores of the company “SEMPAL S.R.L.” from the district of San Jeronimo, province of Andahuaylas and Region Apurimac in Peru. Crop yields averaged 20 t/ha, and native potatoes selected according to weight and size were used, which were of the third category (tubers between 31–60 g and 71–90 mm) and fourth category (tubers smaller than 30 g and smaller than 70 mm). 

All other reagents and supplies used in this research were of analytical grade and were appropriately used in the laboratory, including the quercetin reagent (Sigma Aldrich, St. Louis, MO, USA), AlCl_3_ reagent (Sigma Aldrich, St. Louis, MO, USA), Folin solution (Merck, Darmstadt, Germany), Na_2_CO_3_ solution (Spectrum, NB, Canada), gallic acid reagent (Merck, Darmstadt, Germany), DPPH reagent (HIMEDIA, Mumbai, India), Trolox reagent (Sigma Aldrich, St. Louis, MO, USA), ABTS reagent (Sigma Aldrich, St. Louis, MO, USA), and potassium persulfate K_2_S_2_O_8_ (Biolab, Argentina).

**Figure 6:**
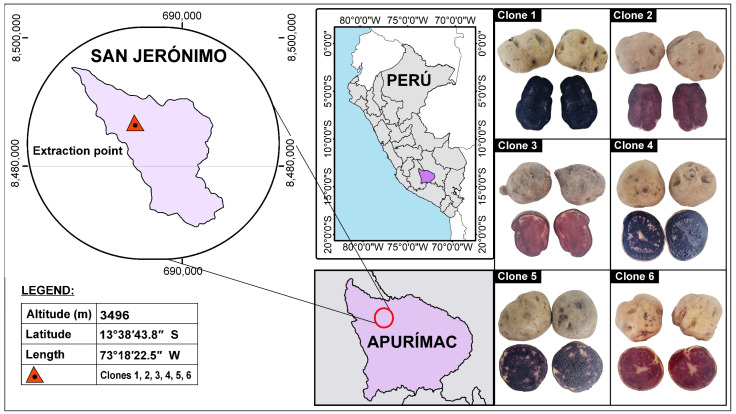
Pigments in native potato clones.

For the selection and classification of the native potato clones in the crop field, the generation of the parents, the location, the year, and the number of experiments used to obtain the new clones were taken into consideration, which were finally chosen based on the best results in terms of production yield and content of bioactive compounds. Figure 6 shows the pigments of the native potato clones used in a geographic information system.

### 3.2. Obtaining Extract from the Selected Native Potato Clone

A total of 150 g of native potatoes were manually crushed in an agate mortar; the obtained mass was mixed with 80% ethanol (2:50 *w/v* ratio) and stirred for 24 h. The mixture was then sonicated with a high-intensity ultrasonic processor (Model VCX 750, Sonics & Materials Inc., New Town, CT, USA) at a frequency of 20 KHz and at a 30% amplitude for 10 min and then centrifuged (TDL-5M model, BIORIDGE, Shanghai, China) at 4000 RPM for 5 min. The supernatant obtained was used for nanoencapsulation.

### 3.3. Nanoencapsulation of Extracts of the Selected Native Potato Clone

A mixture of 90% maltodextrin and 10% gum arabic (*w*/*w*) was used to prepare the encapsulant. A total of 10 mL of aqueous solution of the encapsulant was prepared at 30% (*w*/*v*) and left to stir at 800 rpm for 24 h. After that, 10 mL of the extract previously obtained at a concentration of 2.5% (*w*/*v*) was included in the encapsulation solution. The resulting mixture was homogenized in an ultraturrax (Daihan, HG-15D, Gang-Won-Do, South Korea) at 7500 RPM for 2 min. The proportions above were kept constant for all experiments.

For the nanoencapsulation, a central composite rotatable design (CCRD) was used, considering the inlet temperature (96 and 116 °C) and airflow (120 and 150 L/h) as independent variables, obtaining a 2^2^ factorial design with four central points and four axial points (alpha value of 1.41 for the CCRD), which were experimented in a nano spray dryer B-90 (BÜCHI Labortechnik AG, Flawil, Switzerland) using the smallest nebulizer (4 µm).

The above design was optimized using the response surface methodology (RSM), considering the yield of encapsulation, hygroscopicity, water activity (Aw), moisture, antioxidant capacity, encapsulation efficiency (EE), phenolic compounds, and flavonoids as dependent variables. This is because of preliminary studies; it was observed that the inlet temperature and airflow significantly influenced the properties mentioned before.

The regression analysis and the relationship between the variables were developed using the following second-order polynomial equation.

(1) $$Y=\beta_{0}+\beta_{A}X_{A}+\beta_{B}X_{B}+\beta_{A,B}X_{A}X_{B\;}+\beta_{A,A}X_{A}^{2}+\beta_{B,B}X_{B}^{2}$$

where *Y* is the response variable, *β*_0_ is the constant, *β_A_* and *β_B_* are the linear coefficients, *β_A,B_* is the interaction coefficient, and *β_A,A_* and *β_B,B_* are the quadratic coefficients for the input temperature variables (*X_A_*) and airflow (*X_B_*).

### 3.4. Yield of Encapsulation

The initial mass of the core, the encapsulant, and the final mass of the nanocapsules were recorded and calculated according to the following relation:

(2) $$\%Y=\left(\frac{\mathrm{mi}}{\mathrm{mf}}\right)\times 100$$

where %*Y* is the yield of encapsulation, mi is the initial mass of the core and encapsulant, and mf is the final mass of the nanocapsules [31,35,37].

### 3.5. Hygroscopicity

Hygroscopicity was determined by placing 200 mg of nanocapsules in an airtight container with a saturated NaCl solution (75% relative humidity) at room temperature for up to 7 days. After this time, the final mass of the sample was recorded, and the calculations were carried out considering the following relation.

(3) $$\%I=\left(\frac{m2-m1}{m1-m0}\right)\times 100$$

where %I is the hygroscopicity, m0 is the mass of the petri dish without contents, m1 is the mass of the petri dish + the nanocapsules, and m2 is the final mass of the sample + the petri dish after 7 days [31,35,37].

### 3.6. Water Activity

Samples were analyzed at a temperature of 25 °C in a previously calibrated HygroPalm23-AW portable water activity determinator (Rotronic brand, Bassersdorf, Switzerland) [31,35,37].

### 3.7. Moisture

The methodology described by AOAC 950.10 was used. A total of 2 g of each sample was placed in a forced convection oven FED 115 (BINDER, Tuttlingen, Germany) at 105° until a constant weight was achieved. The initial and final mass of each sample was recorded for the calculations [75,76].

### 3.8. Antioxidant Capacity DPPH and ABTS

Experiments were performed using DPPH (2,2-Diphenyl-1-Picrylhydrazyl) and ABTS+ (2,2′-azino-bis-3-ethylbenzothiazoline-6-sulfonic acid) free radicals. The Trolox (6-hydroxy-2,5,7,8-tetramethylchroman-2-carboxylic acid) reagent was used for the calibration curve. Methanolic extracts were prepared with 500 mg of the sample and 20 mL of 80% methanol, protected from darkness, and kept at room temperature for 24 h.

For the DPPH method, the UV spectrophotometer was brought to zero with methanol. The diluted DPPH solution was adjusted to 1.1 ± 0.02 at a wavelength of 515 nm. Then, to 150 µL of the sample extract, 2850 µL of the diluted DPPH solution was added and allowed to react in test tubes protected from light for 15 min at room temperature. At the same time, a blank was prepared with 150 µL of methanol. Readings were taken at 515 nm, and the results were expressed as µmol ET/g dry sample.

For the ABTS+ method, the radical was prepared with 250 µL of 2.45 mmol K_2_S_2_O_8_ and 25 mL of 7 mmol ABTS and allowed to react in the dark at room temperature for 16 h. The ABTS+ solution was adjusted to 0.7 ± 0.02 at an absorbance of 734 nm. To a total of 0.3 mL of methanolic extract, 2.7 mL of ABTS+ was added, leaving them to react for 15 min; a blank was also prepared with 0.3 mL of methanol. Readings were carried out at 734 nm, and the results were expressed in µmol ET/g dry sample [35,37,68,77].

### 3.9. Encapsulation Efficiency

Encapsulation efficiency was calculated based on the initial content of phenolic compounds of the native potato extract and then nanoencapsulated with maltodextrin and gum arabic, according to the following relation.

(4) $$\%\mathrm{EE}=\left(\frac{TPC_{0}}{TPC_{n}}\right)\times 100$$

where %EE is the encapsulation efficiency, *TPC*_0_ is the total phenolic compound content of the native potato ethanolic extract, and $TPC_{n}$ is the total phenolic compound content of the nanocapsules [31,35,37].

### 3.10. Total Phenolic Compounds

The Folin–Ciocalteu methodology was used based on the chromophore reaction of the methanolic extract of the sample with the Folin–Ciocalteu reagent in an alkaline medium (Na_2_CO_3_), and gallic acid was used for the calibration curve. Methanolic extracts were prepared with 500 mg of the sample and 20 mL of 80% methanol, protected from darkness, and kept at room temperature for 24 h.

A volume of 3300 µL of the methanolic extract and 150 µL of 20% Na_2_CO_3_ were mixed with 300 µL of the Folin–Ciocalteu 0.25 N reagent. It was left to react for 15 min under dark conditions and at room temperature. In parallel, a blank was prepared with the same conditions using distilled water instead of the extract. The absorbance readings were taken at 755 nm, and the results were expressed as mg gallic acid equivalent (GAE)/g dry sample [35,37,68,77].

### 3.11. Total Flavonoids

An ethanolic quercetin solution in concentrations from 0.1 to 20 mg/mL was used for the calibration curve. Extracts of the samples were prepared with 500 mg and 20 mL of 80% methanol, protected in darkness, and kept at room temperature for 24 h.

To 90 µL of the extract, 4.81 mL of 80% methanol and 100 µL of 5% AlCl_3_ were added. It was allowed to stand for 10 min at room temperature. Readings were taken at 425 nm, and the total flavonoid content was expressed as mg quercetin equivalent/g dry sample [35,37].

### 3.12. Total Anthocyanins

The differential pH method of Giusti and Wrolstad was used, for which ethanolic extracts were prepared using 20 mL of the extracting solvent (95% ethanol and 1% HCl) and 1 g of the sample, leaving it to react for 24 h. The samples were treated with 0.025 M KCl and 0.4 M C_2_H_3_NaO_2_ buffers, adjusting the pH to 1 and 4.5, respectively.

Readings were performed at the maximum wavelength and 700 nm (Genesys 150, Thermo Fisher Scientific, Waltham, MA, USA), considering the dilution factor previously calculated with the KCl Buffer. The results were expressed on a dry basis as a mg anthocyanin/g sample [68,77].

### 3.13. Color Analysis

The colorimetric values of the samples were determined by the reflectance module of the CR-5 colorimeter (Konica Minolta, Tokyo, Japan) using a Petri dish with a diameter of 30 mm. The results were expressed as the color parameters L*, a*, and b*. The color variation was calculated with the following formula.

(5) $$\Delta E_{ab}^{*}=\sqrt{\Delta L^{*2}+\Delta a^{*2}+\Delta b^{*2}}\;$$

where $\Delta E_{ab}^{*}$ is the color variation, and $\Delta L^{*}$, $\Delta a^{*}$, and $\Delta L^{*}$ are the differences between L*, a*, and b* of the reference and L*, a*, and b* of the comparison [31,35,37].

### 3.14. Analysis by Scanning Electronic Microscopy (SEM)

A Prisma E scanning electron microscope (Thermo Fisher Scientific, Brno, Czech Republic) was used for morphological analysis. Samples were prepared with 12 mm carbon adhesive disks on 12.7 × 8 mm aluminum stubs. The analysis was performed under a low vacuum at a pressure of 0.07 Torr. The micrographs were observed at a magnification of 5000× at a distance of 10 mm [31,35,37].

### 3.15. Particle Size and ζ Potential

Dynamic light scattering (DLS) was used for particle size determination on a Zetasizer ZSU3100 (Malvern Instruments, Worcestershire, UK). For sample preparation, 20 mg of nanocapsules were dispersed in ultrapure water and sonicated for 10 s. Then, measurements were performed with a He–Ne laser at 633 nm and 20 °C using the DTS002 cell.

A ZSU3100 Zetasizer (Malvern Instruments, Worcestershire, UK) with a He–Ne laser was used to determine the ζ potential. For sample preparation, 20 mg of the nanocapsules were dispersed in 50 mL of ultrapure water and sonicated for 60 s. Readings were taken at 25 °C, and the DTS1080 cell was used. The ZS XPLORER software (Malvern Panalytical Ltd., Malvern, UK) was used for data processing [31,35,37].

### 3.16. Analysis by Fourier Transform Infrared Spectroscopy (FTIR)

Spectra were obtained by Fourier Transform Infrared Spectrometry analysis using the Nicolet IS50 FTIR transmission accessory (ThermoFisher, Waltham, MA, USA). Tablets were prepared with 99% KBr and 1% of the sample. Readings were taken in the mid-IR range (4000 to 400 cm^−1^), with 32 scans at 8 cm^−1^ resolution [31,35,37].

### 3.17. Thermal Analysis

A total of 2 mg of the nanocapsules were used for differential scanning calorimetry analysis (DSC2500, TA Instruments, New Castle, DE, USA), with a temperature range of 0 to 250 °C at a heating rate of 10 °C/min in an N_2_ atmosphere. On the other hand, 10 mg of the nanocapsules were used for thermogravimetric analysis (TGA 550, TA Instruments, New Castle, DE, USA), with a temperature range of 20 to 600 °C at a heating rate of 10 °C/min in an N_2_ atmosphere [31,35,37].

### 3.18. Release of Polyphenols in Aqueous Solution

The Folin–Ciocalteu methodology was used, preparing aqueous solutions with 100 mg of the nanoencapsulates in 10 mL of ultrapure water, left at room temperature and in the absence of light. Readings were taken after 0, 6, 24, and 48 h in a UV spectrophotometer (Thermo Fisher Scientific, Waltham, MA, USA) at a wavelength of 755 nm. The findings were expressed as mg GAE/g on a dry basis [35,37].

### 3.19. Statistical Analysis

The Design Expert v9.0 software (Trial version, Stat-Ease Inc., Minneapolis, MN, USA) was used to analyze regression and the relationship between the independent and dependent variables through a second-order polynomial equation. Graphical representation and other statistical tests were performed with the Origin Pro 2023 software (OriginLab Corporation, Northampton, MA, USA).

## 4. Conclusions

Native potato clone 1 was selected, because of its high content of phenolic compounds, flavonoids, and antioxidant capacity, in order to optimize the nanoencapsulation of phenolic extracts through the response surface methodology, obtaining the optimal treatment at an inlet temperature of 120 °C and an airflow of 141 L/h (desirability of 0.86), which allowed minimizing the dependent variables of hygroscopicity, Aw, and moisture, and maximizing the response variables of yield, antioxidant capacity, encapsulation efficiency, phenolic compounds, and flavonoids. Instrumental characterization of the obtained nanocapsules was also carried out. Regarding color, a gain in lightness and reddening was observed due to the white wall materials used. Thermal analysis, SEM analysis, and IR analysis confirmed the encapsulation of the core in the matrices. Furthermore, spherical, smooth, and heterogeneous particles were observed on the nanometer scale, and a negative ζ potential was found. Finally, a release study of the phenolic compounds was carried out, observing the highest value after 12 h (9.86 mg GAE/g), which would allow its use as an additive in food and pharmaceutical products.

## Figures and Tables

**Table 1:** Characterization of native potato clones.

Properties	Phenolic Compounds (mg GAE/g)				Flavonoids (mg Quercetin Equivalent/g)				DPPH (µmol TE/g)				ABTS (µmol TE/g)				Anthocyanins (mg C3G Equivalent/g)			
	$\bar{x}$	±	SD	*	$\bar{x}$	±	SD	*	$\bar{x}$	±	SD	*	$\bar{x}$	±	SD	*	$\bar{x}$	±	SD	*
Clone 1	6.72	±	0.04	a	3.00	±	0.18	a	131.57	±	0.60	a	22.83	±	0.11	a	7.79	±	0.08	a
Clone 2	5.77	±	0.06	b	2.31	±	0.09	b	21.30	±	1.07	bc	21.37	±	0.25	b	6.23	±	0.04	b
Clone 3	4.82	±	0.03	c	2.21	±	0.09	b	20.51	±	1.16	c	20.38	±	0.14	c	6.58	±	0.13	c
Clone 4	6.44	±	0.07	d	2.87	±	0.33	a	22.89	±	1.39	b	21.41	±	0.10	b	7.20	±	0.05	b
Clone 5	4.64	±	0.04	e	1.90	±	0.11	c	20.18	±	0.25	c	20.17	±	0.04	c	5.19	±	0.34	c
Clone 6	4.57	±	0.04	e	1.68	±	0.11	c	15.08	±	1.80	d	17.52	±	0.06	d	2.53	±	0.02	d

$\bar{x}$, arithmetic mean; SD, standard deviation. * Different letters per column indicate significant differences, for *n* = 3.

**Table 2:** Central composite rotatable design treatments and results of the dependent variables.

Run	A	B	Yield	Hygroscopicity	Aw	Moisture	DPPH	EE	Phenolic Compounds	Flavonoids
	°C	L/h	%	%		%	µmol TE/g	%	mg GAE/g	mg Quercetin/g
			$\bar{x}$ ± SD	$\bar{x}$ ± SD	$\bar{x}$ ± SD	$\bar{x}$ ± SD	$\bar{x}$ ± SD	$\bar{x}$ ± SD	$\bar{x}$ ± SD	$\bar{x}$ ± SD
T1	96	120	66.28 ± 0.33	10.86 ± 0.29	0.44 ± 0.01	3.21 ± 0.09	1.11 ± 0.19	71.44 ± 0.29	4.35 ± 0.12	0.94 ± 0.14
T2	116	120	69.91 ± 0.25	8.65 ± 0.25	0.37 ± 0.02	2.63 ± 0.08	8.58 ± 0.13	74.57 ± 0.19	4.54 ± 0.09	1.18 ± 0.25
T3	96	150	66.35 ± 0.29	10.14 ± 0.25	0.42 ± 0.01	3.19 ± 0.06	4.64 ± 0.21	71.69 ± 0.25	4.36 ± 0.12	0.93 ± 0.08
T4	116	150	74.05 ± 0.31	8.09 ± 0.29	0.36 ± 0.01	2.54 ± 0.09	13.01 ± 0.23	74.93 ± 0.23	4.56 ± 0.17	1.20 ± 0.17
T5	92	135	68.96 ± 0.27	12.26 ± 0.19	0.45 ± 0.03	3.19 ± 0.10	1.19 ± 0.12	68.93 ± 0.18	4.19 ± 0.21	0.60 ± 0.25
T6	120	135	74.28 ± 0.24	7.82 ± 0.14	0.35 ± 0.01	2.45 ± 0.07	28.38 ± 0.14	89.26 ± 0.21	5.43 ± 0.19	2.18 ± 0.14
T7	106	114	67.00 ± 0.25	9.25 ± 0.19	0.39 ± 0.02	3.14 ± 0.08	5.42 ± 0.13	71.55 ± 0.24	4.35 ± 0.14	0.99 ± 0.10
T8	106	156	68.21 ± 0.26	8.10 ± 0.22	0.38 ± 0.01	2.64 ± 0.07	7.45 ± 0.12	74.08 ± 0.26	4.51 ± 0.13	1.15 ± 0.08
T9	106	135	69.15 ± 0.21	8.49 ± 0.19	0.37 ± 0.04	2.98 ± 0.09	6.33 ± 0.17	73.73 ± 0.23	4.49 ± 0.20	1.13 ± 0.07
T10	106	135	69.60 ± 0.22	8.60 ± 0.14	0.38 ± 0.01	2.85 ± 0.08	6.90 ± 0.13	73.60 ± 0.19	4.47 ± 0.18	1.14 ± 0.12
T11	106	135	69.10 ± 0.27	8.48 ± 0.12	0.38 ± 0.02	2.99 ± 0.07	6.40 ± 0.15	73.82 ± 0.28	4.49 ± 0.21	1.13 ± 0.13
T12	106	135	69.78 ± 0.26	8.61 ± 0.16	0.37 ± 0.01	2.84 ± 0.04	6.81 ± 0.16	73.58 ± 0.21	4.48 ± 0.08	1.15 ± 0.11

Where A is the inlet temperature and B is the airflow, for *n* = 3.

**Table 3:** ANOVA of the central composite rotatable design for each dependent variable.

Parameter	Yield	Hygroscopicity	Aw	Moisture	DPPH	EE	Phenolic Compounds	Flavonoids
$\beta_{A}$	44.52 *	13.86 *	0.0092 *	0.65 *	366.90 *	153.19 *	0.57 *	0.94 *
$\beta_{B}$	4.40 *	1.05 *	0.0003 *	0.083 *	14.73 *	2.18	0.008	0.007
$\beta_{A,B}$	4.14 *	0.0064	3.6 × 10^−5^	0.0012	0.20	0.0030	2.5 × 10^−5^	0.0002
$\beta_{A,A}$	6.31 *	3.77 *	0.0014 *	0.0092	62.45	25.44	0.094	0.045
$\beta_{B,B}$	6.75 *	0.042	0.0003 *	4.48 × 10^−5^	7.43	8.70	0.031	0.038
*p*-value model	0.0006	0.0002	<0.0001	0.004	0.03	0.11	0.11	0.12
*p*-value (lack of fit)	0.06	0.007	0.017	0.22	0.0002	<0.0001	<0.0001	<0.0001
R^2^	0.96	0.97	0.99	0.91	0.82	0.71	0.71	0.70

$\beta_{A}$ and $\beta_{A,A}$: linear and quadratic inlet drying temperature; $\beta_{B}$ and $\beta_{B,B}$: linear and quadratic air flow; $\beta_{A,B}$: interaction between inlet drying temperature and air flow; R^2^: coefficient of determination; ∗ significance at *p* = 0.05.

**Table 4:** Estimates of the second-order polynomial regression coefficients for each response.

Regression Coefficient	Yield	Hygroscopicity	Aw	Moisture	Antioxidant Capacity	EE	Phenolic Compounds	Flavonoids
*β*_0_	163.39	123.80	3.33	0.67	211.14	156.40	9.63	1.11
*β_A_*	−2.81 *	−1.82 *	−0.04 *	0.07 *	−6.24 *	−3.87 *	−0.24 *	−0.15 *
*β_B_*	0.58 *	−0.15 *	−0.01 *	0.009 *	1.24	1.43	0.09	0.09
*β_AB_*	0.007 *	0.0003	0.00002	−0.0001	0.002	0.0002	0.00002	0.00005
*β_A_^2^*	0.01 *	0.008 *	0.00015 *	−0.0004	0.032	0.02	0.001	0.0009
*β_B_^2^*	−0.004 *	0.0004	0.00003 *	−0.00001	−0.005	−0.005	−0.0003	−0.0004

A, inlet temperature and B, airflow. * Evaluated through at least 5% significance.

**Table 5:** Optimal parameters of the response variables.

Variable	Experimental Range		Optimal Value	Desirability
Independent variables	Low	High		
A: Inlet temperature (°C)	96	116	120	0.86
B: Air flow (L/h)	120	150	141	
Dependent variables	Low	High	Experimental value	Predicted value
	($\bar{x}$ ± SD)	($\bar{x}$ ± SD)	($\bar{x}$ ± SD)	
Yield (%)	66.28 ± 0.33	74.28 ± 0.24	73.59 ± 0.18	75.37
Hygroscopicity (%)	7.82 ± 0.14	12.26 ± 0.19	6.82 ± 0.13	8.11
Water activity	0.35 ± 0.01	0.45 ± 0.03	0.35 ± 0.01	0.35
Moisture (%)	2.45 ± 007	3.21 ± 009	2.55 ± 0.11	2.39
Antioxidant capacity (µmol TE/g)	1.11 ± 0.19	28.38 ± 0.14	23.05 ± 0.15	22.86
Encapsulation efficiency (%)	68.93 ± 0.18	89.26 ± 0.21	81.80 ± 0.12	83.86
Phenolic Compounds (mg GAE/g)	4.19 ± 0.21	5.43 ± 0.19	4.61 ± 0.17	5.10
Flavonoids (mg quercetin/g)	0.6 ± 0.25	2.18 ± 0.14	1.70 ± 0.16	1.79

$\bar{x}$, arithmetic mean; SD, standard deviation, for *n* = 3.

**Table 6:** Color parameters of the nanocapsules obtained.

Treatments	L*			a*			b*			ΔE*_ab_			Referential Color
	$\bar{x}$	± SD	*	$\bar{x}$	± SD	*	$\bar{x}$	± SD	*	$\bar{x}$	± SD	*	
T1	83.60	0.41	ad	7.49	0.18	a	−1.61	0.03	a	66.79	0.39	ac	
T2	84.55	0.39	b	7.35	0.24	ab	−1.55	0.01	ag	67.73	0.37	b	
T3	85.00	0.43	b	6.80	0.19	c	−1.19	0.02	b	68.14	0.41	b	
T4	84.54	0.62	b	7.49	0.34	a	−1.05	0.03	c	67.75	0.59	b	
T5	84.69	0.28	b	6.26	0.13	d	−1.33	0.02	d	67.79	0.27	b	
T6	83.38	0.08	a	7.20	0.06	b	−0.68	0.03	e	66.58	0.07	a	
T7	82.09	0.51	c	6.68	0.06	ae	−1.44	0.01	f	65.23	0.51	d	
T8	84.29	0.15	bd	7.42	0.04	ab	−1.53	0.03	g	67.47	0.15	bc	
T9	83.21	0.47	a	6.66	0.05	ce	−1.43	0.09	f	66.34	0.47	a	
T10	83.18	0.55	a	6.51	0.09	de	−1.50	0.03	fg	66.30	0.55	a	
T11	83.20	0.47	a	6.67	0.05	ce	−1.44	0.09	f	66.34	0.47	a	
T12	83.18	0.55	a	6.51	0.08	de	−1.50	0.03	fg	66.30	0.54	a	
Toptimal	82.39	0.42	c	5.76	0.07	f	−1.85	0.06	h	65.45	0.01	d	

$\bar{x}$, arithmetic mean and SD, standard deviation. * Different letters per column indicate significant differences, evaluated through at 5% significance for *n* = 3.

**Table 7:** Results of particle size and ζ potential in nanocapsules.

Treatments	Particle Size (nm)			ζ Potential (mV)		
	$\bar{x}$	±SD	*	$\bar{x}$	±SD	*
T1	143.58	0.01	a	−25.02	0.01	a
T2	136.33	0.03	b	−28.30	0.07	b
T3	159.37	0.02	c	−33.16	0.03	c
T4	134.02	0.03	d	−22.79	0.08	d
T5	133.09	0.02	e	−31.12	0.02	e
T6	148.12	0.03	f	−24.38	0.01	f
T7	156.87	0.04	g	−33.57	0.06	g
T8	165.13	0.05	h	−26.38	0.05	h
T9	156.40	0.02	i	−27.51	0.03	i
T10	153.10	0.03	j	−23.85	0.02	j
T11	155.30	0.04	k	−29.39	0.03	k
T12	156.10	0.03	l	−28.93	0.05	l
Optimal	148.14	0.02	f	−29.41	0.04	k

$\bar{x}$, arithmetic mean and SD, standard deviation. * Different letters per column indicate significant differences, evaluated through at 5% significance for *n* = 3.

## Data Availability

Data are available in this article.

## Sample Availability

Not available.
